# Paternal Age and Risk of Autism in an Ethnically Diverse, Non-Industrialized Setting: Aruba

**DOI:** 10.1371/journal.pone.0045090

**Published:** 2012-09-11

**Authors:** Ingrid D. C. van Balkom, Michaeline Bresnahan, Pieter Jelle Vuijk, Jan Hubert, Ezra Susser, Hans W. Hoek

**Affiliations:** 1 Child and Adolescent Psychiatry Clinic, Oranjestad, Aruba; 2 Jonx Department of Youth Mental Health, Lentis Psychiatric Institute, Groningen, The Netherlands; 3 Rob Giel Research Center, Department of Psychiatry, University Medical Center of Groningen, University of Groningen, Groningen, The Netherlands; 4 Mailman School of Public Health, Columbia University, New York, New York, United States of America; 5 New York State Psychiatric Institute, New York, New York, United States of America; 6 Department of Clinical Neuropsychology, VU University, Amsterdam, The Netherlands; 7 Child and Youth Health Services, Department of Public Health of Aruba, Oranjestad, Aruba; 8 College of Physicians and Surgeons of Columbia University, New York, New York, United States of America; 9 Parnassia Bavo Psychiatric Institute, The Hague, The Netherlands; 10 Department of Psychiatry, University Medical Center Groningen, University of Groningen, Groningen, The Netherlands; University of Queensland, Australia

## Abstract

**Objective:**

The aim of this study was to examine paternal age in relation to risk of autism spectrum disorders (ASDs) in a setting other than the industrialized west.

**Design:**

A case-control study of Aruban-born children (1990–2003). Cases (N = 95) were identified at the Child and Adolescent Psychiatry Clinic, the only such clinic in Aruba; gender and age matched controls (N = 347) were gathered from public health records. Parental age was defined categorically (≤29, 30–39, 40–49, ≥50y). The analysis was made, using conditional logistic regression.

**Results:**

Advanced paternal age was associated with increased risk of ASDs in offspring. In comparison to the youngest paternal age group (≤29y), risk of autism increased 2.18 times for children born from fathers in their thirties, 2.71 times for fathers in their forties, and 3.22 thereafter.

**Conclusion:**

This study, part of the first epidemiologic study of autism in the Caribbean, contributes additional evidence, from a distinctive sociocultural setting, of the risk of ASD associated with increased paternal age.

## Introduction

Major studies showing that advanced paternal age elevates risk of autism in offspring have been conducted in predominantly high-income countries (the United States (California), Denmark, Israel, Western Australia, Sweden, the Netherlands, the United Kingdom) [1]–[6].

The mechanisms underlying the association between advanced parental age and autism risk are not yet fully understood. The leading hypothesis is that with advancing paternal age, de novo genomic alterations and/or changes in gene expression regulation levels increase the risk of autism [7], [8]. Alternatively, delayed parenthood could reflect sub threshold autistic traits in individuals leading them to delay parenting [9], [10]. There are also suggestions that sociocultural determinants of age at parenting may better explain the finding. Sociocultural factors which influence age at parenting differ across countries and include factors such as immigration, access to family planning services, educational attainment, and socioeconomic status [11]–[14]. The significance of these sociocultural factors is difficult to evaluate due to the lack of sociocultural diversity of the major studies to date. Studies of autism in a greater diversity of settings are underway or have recently been published [15], [16]. Among the first of these was a prevalence study of treated autism spectrum disorders in Aruba [17]. Our aim in the current study was to examine the hypothesis that advanced paternal age increases risk of autism in the non-industrial, ethnically diverse setting of Aruba.

## Methods

### Area and population

Aruba is a Caribbean island 17 miles off the coast of Venezuela (population 90,506 in 2000) The native-born population of Aruba is predominantly of Amerindian (Arawak), Dutch, and Spanish ancestry [18]. In conjunction with an economic transition in the 1990s, Aruba absorbed a large number of immigrants. Since 2000, immigrants have constituted at least 30% of the population [19]. Although social distinctions based on race may exist, these are nowhere documented, and race is officially considered a continuously distributed trait. During the 1990s health insurance was nearly universal for legal residents; in 2001 access to health care in Aruba improved further with the introduction of mandatory health insurance. All children of legal residents are entitled to health care [17].

### Study Design

This study is a population-based case-control study using clinic and public health records. The sampling frame includes all births in Aruba between 1990 and 2003 recorded in the Aruban public health records. Autism in children born between 1990–1999 had previously been identified in the Aruba Autism Project, a prevalence study of Autism Spectrum Disorders (ASDs) in Aruba [17]. This earlier prevalence study was extended to include children born from 1990 to 2003, from clinic records of assessments recorded until January 1, 2006. Controls were selected from the public health records (well-baby clinics records and adolescent health preventative clinic records) matching on date/month/year of birth and gender.

### Case identification

Records from the Aruba Child and Adolescent Psychiatry Clinic, the first and only child and adolescent psychiatry service on the island, were systematically screened for diagnosed and suspected cases of ASD in children born in Aruba from 1990 to 2003, extending the birth years investigated in the prevalence study of treated ASDs in Aruba [17]. Standardized chart abstractions were conducted on all potential cases, including children with a clinic ASD diagnosis, or a working diagnosis [20] of ASD; abstracts were reviewed and a study diagnosis was assigned based on chart evidence of symptoms in accordance with DSM-IV symptom criteria. Autism Spectrum Disorders were defined to include Autistic Disorder, Asperger's Disorder, and Pervasive Developmental Disorder Not Otherwise Specified. Eligibility for inclusion in the analysis was based wholly on chart review, and not on standing clinic diagnosis. In total, 101 cases of ASDs were identified by these methods and included; 23 of these cases were validated in a previous study (see Van Balkom et al., 2009 [17] for a more complete description).

### Control identification

A minimum of three and a maximum of five controls, matched to each subject classified with an ASD for date of birth and gender, were randomly selected from the public health records. With these methods 469 controls were identified for the 101 ASD subjects.

### Covariate information

Data on controls were abstracted from the centralized computer records of the Aruban public health clinics, which serve all Aruban children from infancy to age 10 to 11 years. Public health clinic files for selected controls were retained in one of two locations (the centralized archive or home clinic) depending upon birth years. Clinic files include immunization history, visit notes, and in most instances parental characteristics including parents' place of birth, date and/or year of birth, maternal parity, and parental occupation. Parental characteristics were abstracted for each control using a standardized abstraction form. Only anonymized data were extracted from the clinic records. A similar abstraction process for parental characteristics of cases was carried out.

Characteristics of the study sample are shown in Table 1.

**Table 1:** Characteristics of the study sample.

*Variable*		*Controls N (%) N = 347*	*Cases N (%) N = 95*	*Total N (%) N = 442*
***Paternal Age***	***≤29***	149 (42.9)	23 (24.2)	172 (38.9)
	***30–39***	158 (45.5)	55 (57.9)	213 (48.2)
	***40–49***	36 (10.4)	15 (15.8)	51 (11.5)
	***≥50***	4 (1.2)	2 (2.1)	6 (1.4)
***Maternal Age***	***≤29***	211 (60.8)	42 (44.2)	253 (57.2)
	***30–39***	130 (37.5)	47 (49.5)	177 (40.0)
	***40–49***	6 (1.7)	6 (6.3)	12 (2.7)
***Preterm birth***	***Yes***	29 (8.4)	10 (10.5)	39 (8.8)
	***No***	304 (87.6)	76 (80.0)	380 (86.0)
	***Missing***	14 (4.0)	9 (9.5)	23 (5.2)

Six of the cases classified as ASD for this study were excluded due to missing data on mother and/or father's age along with their 26 matched controls. An additional 96 controls were excluded due to missing data on maternal and/or paternal age. The final sample consisted of 95 cases and 347 controls.

### Variables

Paternal and maternal age, the primary confounder [6], [21], were defined as categorical variables. Parental age was categorized in 10 year increments, resulting in four categories of paternal age (≤29 =  age group 1 (reference category), 30–39 =  age group 2, 40–49 =  age group 3, and ≥50 =  age group 4), and three categories of maternal age as there were no mothers older than 49 years (≤29 =  age group 1 (reference category), 30–39 = age group 2, and 40–49 =  age group 3). Three additional covariates were identified for inclusion as possible confounders: low birth weight [13], [23], [24], preterm birth [13], [22]–[24] and parental immigrant status [4], [25]. Low birth weight was defined as <2,500 grams (N = 11), and preterm birth as ≤37 weeks pregnancy (N = 39). Parental place of birth was classified as Aruba (A) and not Aruba (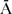); from this four categories of combined parental place of birth were defined, i.e. A_FA_A_MO_ (reference category, N = 207), A_FA_ 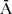
_MO_ (N = 90), 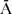
_FA_A_MO_ (N = 47), 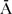
_FA_ 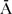
_MO_ (N = 74); for 24 children the birthplace of the parents was unknown. Neither low birth weight, nor parental immigrant status were associated with ASD in bivariate analysis, and were therefore dropped from the models.

### Analysis

The data were analyzed using STATA version 9. Conditional logistic regression was used for matched case-control groups with STATA's “clogit” command to examine paternal age effects of increased risk in ASDs in offspring unadjusted, while controlling for maternal age effects, and while controlling for maternal age and preterm birth effects, as well as the interaction effect beteen maternal age and paternal age.

### Confidentiality

In keeping with Dutch medical ethical guidelines for the conduct of record review studies personal information was treated confidentially. Only the treating child psychiatrist and the research psychiatrist had access to the medical charts. Data were entered into a statistical database without identifying information. Informed consent was not applicable. The study was approved by the Ministry of Health of Aruba.

## Results

Mean paternal age in cases was 33.5 (sd = 6.8), and in controls 31.1 (sd = 7.1) years; mean maternal age in cases was 30.2 (sd = 5.7), and in controls 27.6 (sd = 5.6) years.

Advanced paternal age was associated with increased risk of ASDs in offspring (Table 2). In comparison to the youngest paternal age group (≤29), the risk of autism increased significantly to 2.18 times (p = .004) for children with fathers in their thirties, and to 2.71 times (p = .01) in their forties. Adjusting for maternal age, paternal age effects were significant for fathers in their thirties, compared to younger fathers. However effects were rendered non-significant for other paternal age groups. When adjusting for confounding variables maternal age and preterm birth, fathers in their thirties (OR = 2.16; p = .02) and forties (OR = 2.67; p = .04) have a significantly increased risk for ASDs in their offspring compared to the reference group. No significant interaction effect was found between paternal age and maternal age (p = .55).

**Table 2:** Odds ratios for paternal age adjusted for maternal age and preterm birth.

*Models♦*	Model 1		Model 2		Model 3	
*Variable*	*OR* (*95% CI*)	*p*	*OR* (*95% CI*)	*p*	*OR* (*95% CI*)	*p*
***Paternal Age*** *						
30–39	2.18 (1.29,3.72)	.004	1.85 (1.03,3.29)	.04	2.16 (1.15,4.04)	.02
40–49	2.71 (1.27,5.78)	.01	2.00 (0.87,4.61)	.10	2.67 (1.07,6.68)	.04
≥50	3.22 (0.55,18.68)	.19	2.24 (0.36,13.87)	.39	2.38 (0.37,15.40)	.36
***Maternal Age*** *						
30–39			1.41 (0.83,2.40)	.20	1.51 (0.86,2.67)	.15
40–49			3.39 (0.92,12.49)	.07	3.45 (0.84,14.10)	.09
***Preterm birth •***						
≤37 weeks					1.08 (0.50,2.37)	.84

♦ =  Model 1: Paternal age; Model 2: Paternal age + Maternal age; Model 3: Paternal age + Maternal age + Preterm birth.

* =  reference category: age group ≤29 years; •  =  reference category: “not preterm birth”.

## Discussion

In this case-control study in a total population Aruban birth cohort (1990–2003) we found that advanced paternal age, in comparison to young paternal age (≤29), was associated with a greater than two-fold increased risk of ASDs in offspring. Adjusting for both maternal age and preterm birth had little impact on the finding. These results are consistent with other reports from western countries [1]–[6], and fall within the range of effects reported in a recent meta-analysis [4].

The leading explanations for increasing risk with advancing paternal age are genetic mutations/cytogenetic abnormalities known to increase with age [26], [27], or age associated methylation differences [28]. The leading alternative explanation is that subthreshold autistic traits in the fathers, associated with delayed or late parenting, elevates the risk of autism. Support for this hypothesis is based in part on evidence of social and communication impairments in parents and siblings of autistic probands, referred to as the broader autism phenotype (BAP) [29].

Attempts to discriminate between the two major alternatives (mutation/epigenetics versus broader autism phenotype self selection) have adopted different strategies. The first strategy is the most direct, and examines parental characteristics and reproductive age in multiplex families. Using this strategy, Puleo and colleagues [10] found that no major dimension of the broader autism phenotype increased with age at paternity.

A second strategy, also focused within affected families, reasons that an association of paternal age and risk of autism within affected families argues against a primary role for the self-selection hypothesis. Hultman et al. [4] examined paternal age effects in sibships with one or more autistic children, and one non-autistic sibling, and found that the association was observed in first born and subsequent births. Stratifying on age at birth of first child, increasing risk with advancing paternal age was reported for all but one strata. Parner et al. [30] included a sibling subcohort analysis which adjusted for common genetic and environmental factors, and found that the parental age association persisted, and increased risk of ASD could not be explained by these factors.

The study in Aruba reflects a third strategy. This approach, focused on culturally distinct environments, reasons that in different cultural circumstances individuals selected into delayed parenting will differ. For example, it is likely that social, cultural, and ethnic influences on age of reproduction in Aruba are affected by the transitional economy and the rapid influx of immigrants, changing the meaning of older ages at parenting. If paternal age effects are consistent across cultures, this would argue for an age related, rather than BAP related effects.

In Aruba, we found a two to three-fold increased risk of autism in offspring associated with advancing paternal age. These findings add to the line of evidence demonstrating consistent paternal age effect in culturally dissimilar environments. Two case control studies in non-western countries – Iran and China – have recently been published [15], [16]. In the Iranian study, controls were matched to cases on parental education, birth order, sex, consanguinity, urbanism and province. In matched analysis, fathers age 35–39 had nearly a twofold increased risk, and fathers 40+ a 2.58 times increased risk compared to fathers aged 25–29. A small case control study in Tianjin, China, reported a significant advanced paternal age (>30) effect, associated with increased risk of 2.63 adjusting for sex and birth year. Maternal age effects were nonsignificant. It is notable that in both Iran and China, arranged marriages are common.

### Strengths and limitations

Major strengths of the study include access to rigorously defined cases arising in the total population of Aruban births 1990–2003, ascertainment through the only child psychiatry clinic within a well-established universal health care system, and accurate enumeration of the population at risk through the population registry.

Nonetheless, the limitations of the present study also need to be considered. First, the small sample size is an inherent limitation to the study of this population. In addition, the decrease in the effect size for paternal age when maternal age is added to the model, may reflect the difficulty of fully separating paternal and maternal age effects; the instability of the confidence intervals clearly reflects the limited sample size.

A second limitation pertains to the record-based methodology. Findings with respect to assigning a study diagnosis, as in any record-based study, are usually limited by the absence of in-person standardized research interviews and direct clinical assessments of the study classified cases. However, in a prior validation study (N = 30; 24 cases and 6 controls) we reported that 23 of the 24 cases were confirmed. Since these 23 cases have been included in the present study the diagnosis of 24% of present study cases was validated [17].

## Conclusion

The study contributes additional evidence from a distinctive sociocultural setting, to the literature on the relationship between paternal age and risk of ASDs, and it emphasizes the importance of replicating these findings across environments since increased paternal age may encapsulate both biological and sociocultural risk factors for adverse neurodevelopmental outcomes in offspring. Taken together with findings from other studies employing distinct research designs, these findings suggest that the paternal age effect is not explained by a selection effect in which fathers with autistic traits preferrentially delay parenting.
